# Validation of the Aarhus periacetabular osteotomy database

**DOI:** 10.1007/s00402-025-05978-7

**Published:** 2025-08-28

**Authors:** Lisa Tønning, Frederik Foldager, Josefine Larsen, Pia Kristensen, Inger Mechlenburg, Kjeld Søballe, Stig Jakobsen

**Affiliations:** 1 Department of Orthopedic Surgery, Aarhus University Hospital, Aarhus, Denmark; 2 Department of Clinical Medicine, Aarhus University, Aarhus, Denmark; 3 Department of Public Health - Sport, Aarhus University, Aarhus, Denmark

**Keywords:** Hip dysplasia, Periacetabular osteotomy, Validation, Danish National patient registry

## Abstract

**Background:**

Periacetabular osteotomy (PAO) is the preferred surgical treatment for hip dysplasia. In Denmark, patients undergoing PAO at two orthopaedic departments are registered in a disease registry, the Aarhus PAO-database. This study aimed to validate the Aarhus PAO-database by assessing the registration completeness compared to the Danish National Patient Registry (DNPR).

**Materials and methods:**

Patients registered in the Aarhus PAO-database were compared to patients identified in DNPR as having undergone PAO for hip dysplasia. Further, a random selection of 20 PAO procedures from each registry per year (2014–2021) was validated by comparing the information from the registries to the hospital’s electronic medical records.

**Results:**

Between 2014 and 2021, 1144 PAO procedures were registered in the Aarhus PAO-database and 1150 in DNPR. The overall registration completeness was 94.7% (95% CI: 93.3;95.9) with 1116 PAO proceduresincluded in both datasets. The diagnosis and surgery procedure were verified as hip dysplasia and PAO for all randomly selected patients, and almost all discrepancies were resolved (> 95%), using the medical records.

**Conclusion:**

The Aarhus PAO-database has effectively captured all patients who underwent PAO for hip dysplasia from 2014 to 2021. It appears to be a valid resource for future research as well as for ensuring and improving the quality of hip dysplasia treatment.

## Introduction

Hip dysplasia is a developmental joint disease, affecting 3.4% of Danish adults [1]. Hip dysplasia is characterised by a shallow oblique acetabulum, ligament laxity, and proximal femur abnormalities leading to insufficient femoral head coverage [2] This insufficient coverage often includes deficient lateral acetabulum coverage, sometimes with anterior or posterior deficiency [3]. Typical symptoms include hip and/or groin pain, altered gait and reduced range of motion, though not all adults with hip dysplasia are symptomatic [4, 5]. Those experiencing pain may undergo periacetabular osteotomy (PAO), a surgery that reorients the acetabulum to increase femoral head coverage [6].

Since 1998, patients undergoing PAO at either Aarhus University Hospital or Mølholm Private Hospital have been registered in the Aarhus PAO-database. This disease registry has been used for research on hip pain, function, quality of life, the familial prevalence of hip dysplasia, complication rates and radiographic measurement’s reliability [7–12]. However, the data has never been validated and the number of missing registrations compared to the Danish National Patient Registry (DNPR) has not been investigated. DNPR is an administrative registry ensuring hospitals are reimbursed for healthcare services and is considered the gold standard for hospital healthcare services [13]. All PAO procedures should be registered in DNPR and validation can be done by comparing DNPR information to the individual patient’s medical records [13, 14].

The first aim of this study was to investigate the registration completeness in the Aarhus PAO-database compared to DNPR. The second aim was to investigate the positive predictive value (PPV) of the diagnosis and surgical procedure from a random sample of 160 patients each from the Aarhus PAO-database and DNPR, compared to their electronic medical records. The third aim was to investigate the PPV of the diagnosis and surgical procedure for patients with discrepancies between the two registries, using electronic medical records.

## Method

In Denmark, the majority of health care services are publicly funded through taxation, ensuring that all residents have broad and equal access to general practitioners and hospital care. A small share of services is provided by private practitioners, who also report to the DNPR [15]. This validation study on the Aarhus PAO-database and was approved by the Legal Office of the Regional Midtjylland Secretariat (journal number 1-45-70-85-22) and registered at the Region of Central Denmark’s internal list of research projects (journal number 1-16-02-46-23).

### Indications for PAO surgery

Indications for PAO surgery during the study period were (i) persistent hip pain and reduced function, (ii) radiographically verified hip dysplasia, defined as having a lateral center edge angle of Wiberg < 25°, (iii) skeletal maturity and (iv) absence of hip subluxation. Contraindications were (i) reduced range of motion, defined as internal rotation ≤ 15° and hip flexion ≤ 110°, (ii) hip osteoarthritis, defined as having a Tönnis grade > 0, (iii) body mass index > 25 and (iv) age > 45 years. The last three contraindications were added in 2016. Since 2004 the minimally invasive transartorial approach has been used when performing PAO at both hospitals [6].

### Data sources

#### The Aarhus PAO-database

The disease registry was created in 2010 and includes prospectively gathered data from patients undergoing PAO at Aarhus University Hospital and Mølholm Private Hospital. Data from patients operated from 1998 to 2010 were stored in paper format and retrospectively entered into the registry in 2014 by a secretary at the department of Orthopedic Surgery at Aarhus University Hospital. The registry contains information on patient demographics, radiological findings, surgery-related information and patient-reported outcomes. Radiological findings and surgical information are collected before and after surgery and entered prospectively into the registry by the orthopaedic surgeon. Patient-reported questionnaires are collected by e-mail preoperatively, 6 months after surgery as well as 2, 5, 10, 15 and 20 years after surgery. The patient-reported questionnaires are emailed to the patients, and all data is stored using the software Procordo v3.0 (Procordo Aps, København, Denmark).

#### The Danish National patient registry

The DNPR is a national registry that has collected data from all Danish hospitals, both public and private, since 1978 [13]. All hospitals, both public and private, are required by Danish law to submit standardised data to the DNPR monthly on all hospital contacts, including surgical procedures and diagnoses [13], which means that the registrations from these two settings can be considered comparable. In DNPR diagnosis are registered using the Internal Classification of Diseases 10th revision (ICD-10) codes, while surgical procedures are registered with NOMESCO Classification of Surgical Procedures (NCSP) codes and hospitals with SHAK codes (Health Care Classification System) [13].

#### Medical record

The electronic medical records in the Central Denmark Region include individual healthcare-related registrations for each hospital visits linked with date, time, department and the healthcare professional. While the DNPR is based on these records, the medical records contain more comprehensive information and notes from the health professionals, making them the gold standard for information regarding diagnosis and treatment [14].

### Study population

#### The Aarhus PAO-database

From the Aarhus PAO-database all patients registered were considered eligible. Exclusion criteria included (i) PAO performed before 2014 or after 2021, (ii) double entry (the second PAO on the same hip were ineligible), (iii) diagnosis of Legg-Calvé-Perthes, (iv) femur osteotomy as the surgical procedure, (v) skeletal immaturity (age < 15 years at surgery) and (vi) medical tourists (foreigners without a Danish social security number).

#### The Danish National patient registry

The 1st of January 2014 was defined as the starting time point, as despite the merger of three hospitals into Aarhus University Hospital in 2011, the SHAK codes for the three hospitals remained active until late 2013.

Patients were identified as having undergone PAO using the NCSP codes “NEK59” (pelvic osteotomy) and “NET49” (correction of pelvis deformity). Patients with ICD-10 code “Q658” (congenital malformation of the hip) as the diagnosis code, were eligible for inclusion. Aarhus University Hospital is in DNPR defined by the SHAK code “6620” and Mølholm Private Hospital has the SHAK code “6010”. In addition, information regarding social security number, department and age at the time of surgery was extracted to exclude patients treated before skeletal immaturity. To avoid extracting unnecessary data, it was decided to only include patients from DNPR where both the diagnosis (hip dysplasia) and the treatment (PAO) had been registered as well as the treating hospital being either Aarhus University Hospital or Mølholm Private Hospital. As not all patients with hip dysplasia undergo PAO, a data extraction based solely on the diagnosis would include many irrelevant patients. The exclusion criteria were thus limited to skeletal immaturity (age < 15 years at surgery).

#### Medical record

From the medical records hip dysplasia was defined as any mention of hip dysplasia as the underlying pathology for symptoms recorded by an orthopaedic surgeon based on a clinical and radiographic assessment. PAO was defined as a surgical description by the treating orthopaedic surgeon identifying PAO as the surgery performed. All information from the medical records was extracted by a single independent researcher (LT), who was not involved in the treatment of these patients, using a standardised form in Research Electronic Data Capture (REDCap) [16, 17].

### Statistical analysis

The results are presented as the number of included and missing patients from the Aarhus PAO-database and the DNPR. The completeness of registration was assessed by calculating the number of PAO procedures (hip dysplasia treated with PAO) registered in both the Aarhus PAO-database and DNPR divided by the number of PAO procedures registered in the Aarhus PAO-database or DNPR, with 95% confidence intervals (95% CI). This was done for the entire period as well as for each year from 2014 to 2021. A sensitivity analysis was done by adding the date of the PAO surgery. In the sensitivity analysis date registration could only differ by one day between the PAO-database and the DNPR to calculate the registration completeness. In addition, a computer-generated random selection of 20 PAO procedures from the Aarhus PAO-database for each year (2014–2021) was validated by comparing the information in the Aarhus PAO-database and DNPR to the information in the electronic medical records. The PPV was calculated as the proportion of diagnoses and procedures in the Aarhus PAO-database and DNPR confirmed by the hospital’s medical records from the patients from the random sample. Additionally, information on diagnosis, surgery, the date of surgery, hip side and hospital, was extracted from the medical records. In cases with discrepancies between the Aarhus PAO-database and DNPR, the PPV was calculated among the PAO procedures with discrepancies, as the proportion of diagnoses and procedures confirmed by the medical records. All data from the medical records was managed using the secure and web-based software platform, Research Electronic Data Capture (REDCap) hosted at Aarhus University [16, 17]. All statistical analysis was performed in Stata version 18.0 (StataCorp LLC, College Station, TX, USA).

## Results

**Fig. 1:**
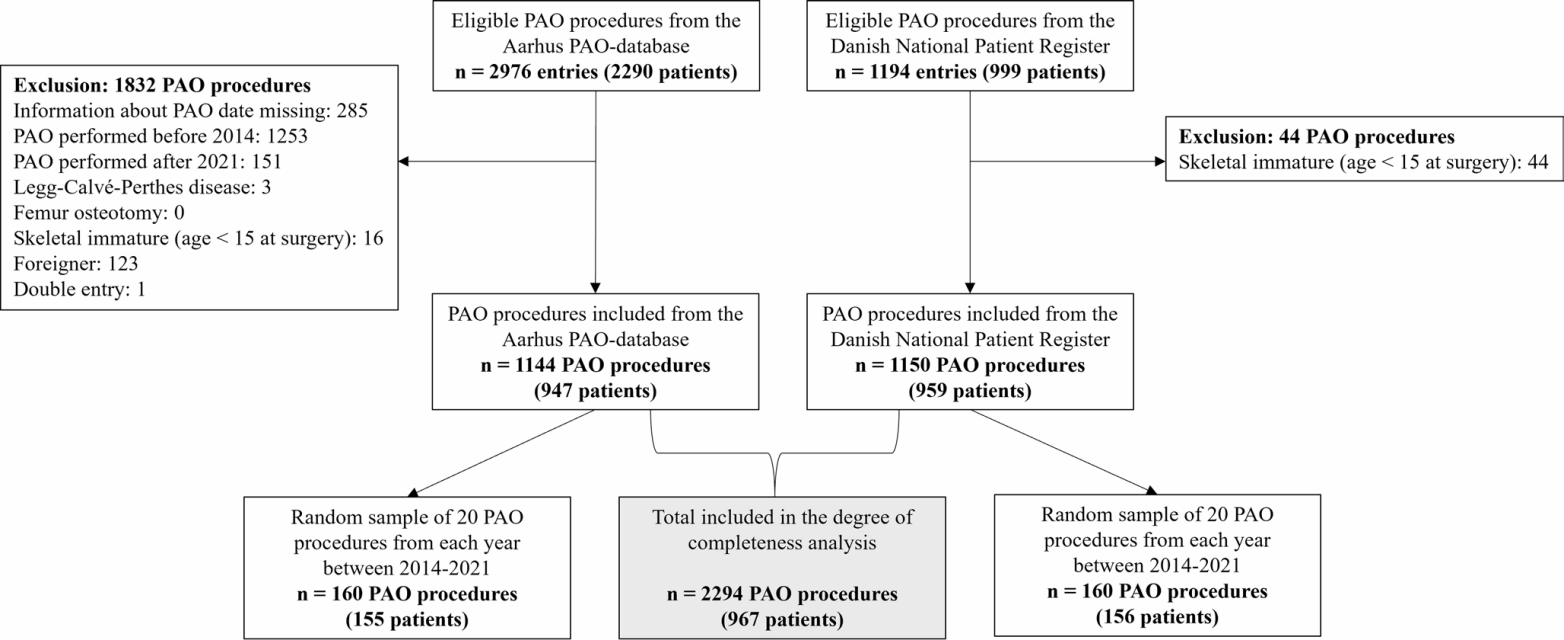
Flow chart of the included hips from the Aarhus periacetabular osteotomy database (Aarhus PAO-database) and the Danish National Patient Register (DNPR)

From the Aarhus PAO-database 2976 PAO proceduresfrom 2290 patients were eligible (Fig. 1). After excluding 1832 PAO proceduresbased on the exclusion criteria, 1144 PAO proceduresfrom 947 patients were included. From the DNPR 1194 PAO proceduresfrom 999 patients were eligible. After excluding 44 PAO proceduresdue to patients being younger than 15 at the time of surgery, 1150 PAO procedures from 959 patients were included. Most PAO procedures were from women and the PAO had been performed at Aarhus University Hospital (Table 1). There were no significant differences between age, sex and hip side between the two datasets. For 18 PAO procedures the hospital information was missing in the Aarhus PAO-database.

**Table 1:** The patient characteristics of all included PAO procedures (hips that had undergone PAO due to hip dysplasia) based on the information collected from the Danish National patient registry (DNPR) and Aarhus PAO-database

Patient characteristic		Both DNPR^a^ and Aarhus PAO-database^b^	DNPR^a^ only	Aarhus PAO-database^b^ only
Number of patients, n (%)		967	959	947
Number of hips, n (%)		1178	1150	1144
	Aarhus University Hospital, n (%)	884 (75.0%)	880 (76.5%)	863 (75.4%)
	Mølholm Private Hospital, n (%)	278 (23.6%)	270 (23.5%)	263 (23.0%)
Women, n (%)		1010 (85.7%)	990 (86.1%)	982 (85.8%)
Age at the time of surgery, mean (SD)		28.8 (9.6)	28.9 (9.6)	28.3 (9.6)
	Age < 18 years, n (%)	97 (8.2%)	94 (8.2%)	97 (8.5%)
	Age 18–40, n (%)	871 (73.9%)	849 (73.8%)	845 (73.9%)
	Age > 45, n (%)	210 (17.8%)	207 (18.0%)	202 (17.7%)
Operation side				
	Right side, n (%)	587 (49.8%)	578 (50.3%)	635 (55.5%)
	Left side, n (%)	482 (40.9%)	463 (40.3%)	509 (44.5%)

DNPR: Danish National Patient Registry, PAO: Periacetabular Osteotomy

^a^Less than 5 PAO procedures from DNPR had missing information on sex and age and 109 missing operations side in DNPR

^b^18 PAO procedures from the Aarhus PAO-database had missing information on hospital

### Completeness of registration

The first aim of this study was to investigate the registration completeness in the Aarhus PAO-database compared to DNPR. There were 1178 PAO proceduresin total and 1116 were included in both datasets (Table 2). 34 PAO procedures (2.9%) were included in DNPR but not in the Aarhus PAO-database, while 28 (2.4%) were included in the Aarhus PAO-database but not in DNPR. When stratified by hospital, 97.3% PAO procedures from Aarhus University Hospital and 92.1% from Mølholm Private Hospital were included in both datasets (Table 2). The overall registration completeness between the Aarhus PAO-database and DNPR was 94.7% (95% CI: 93.3;95.9) and remained consistent over time (Table 3). The sensitivity analysis, allowing a maximum of 1 day difference in the data of surgery between the two registries, showed a registration completeness of 87.1% (95% CI: 85.0;90.0). The median difference between the two registries, in registered date of surgery among patients with more than one days difference was 3 days (interquartile range: 2;99 and range 2;878).

**Table 2:** The number of included and missing PAO procedures (hips that had undergone PAO due to hip dysplasia) in the Aarhus PAO-database and the Danish National patient registry (DNPR)

		Aarhus PAO-database					
		Total		Aarhus University Hospital		Mølholm Private Hospital	
		Included	Missing	Included	Missing	Included	Missing
Danish National Patient Registry	Included	1116 (94.7%)	34 (2.9%)	860 (97.3%)	20 (2.3%)	256 (92.1%)	14 (5.0%)
	Missing	28 (2.4%)	NA	< 5 (0.5%)	NA	8 (2.9%)	NA

18 PAO procedures from the Aarhus PAO-database had missing hospital information. DNPR: Danish National Patient Registry, PAO: Periacetabular Osteotomy

**Table 3:** Completeness of registration of periacetabular osteotomy (PAO) among patients with hip dysplasia in the Aarhus PAO-database compared with the Danish National patient registry (DNPR)

Year	Total, *n*	DNPR, *n* (%)	Aarhus PAO-database, *n* (%)	Both DNPR and Aarhus PAO- database, *n* (%)	Degree of completeness, % (95% CI)
2014–2021	1178	1150	1144	1116 (94.7%)	94.7 (93.3;95.9)
2014	145	144	140	139 (95.9%)	95.9 (91.2;98.5)
2015	171	170	161	160 (93.6%)	93.6 (88.8;96.7)
2016	190	177	187	174 (91.6%)	91.6 (86.7;95.1)
2017	159	155	155	151 (95.0%)	95.0 (90.3;97.8)
2018	129	127	125	123 (95.4%)	95.3 (90.2;98.3)
2019	150	148	146	144 (96.0%)	96.0 (91.5;98.5)
2020	146	143	143	140 (95.9%)	95.9 (91.3;98.5)
2021	88	86	87	85 (96.6%)	96.6 (90.4;99.3)

CI: Confidence Interval. DNPR: Danish National Patient Registry, PAO: Periacetabular Osteotomy

### The randomly selected sample

320 PAO procedures were randomly selected (160 from each registry) and their registered diagnosis and surgical procedure in the Aarhus PAO-database and DNPR were validated using the electronic medical records. All 320 PAO procedures were confirmed to have hip dysplasia and had undergone PAO. The hospital registered in DNPR matched the Aarhus PAO-database for 318 PAO procedures, with a PPV were 0.99 (95% CI: 0.98;1.00). For 67 PAO procedures the PAO date differed between the two datasets and the medical records, with a median difference of 1 day (interquartile range: 1;2) ranging from 1 to 930 days. For 61 PAO procedures the difference was less than a week. The PPV for the date of PAO among the randomly selected PAO procedures was 0.79 (95% CI: 0.74;0.83).

### The discrepancies

There were 62 PAO procedures with a discrepancy between DNPR and the Aarhus PAO-database, ranging from <5 to 16 per year (Table 3). Most of these patients were verified as having hip dysplasia and PAO using the medical records. Fewer than 5 of the 62 PAO procedures with a discrepancy could not be verified, these PAO procedures were either diagnosed with a different condition or had undergone a different surgery than PAO. These procedures were all from DNPR, the PPV was 0.97 (95% CI: 0.89;1.00) overall, and 0.95 (95% CI: 0.82;0.99) for DNPR.

## Discussion

The overall registration completeness was 94.7% (95% CI: 93.3;95.9) between the Aarhus PAO-database and DNPR, meaning that 95% of hips undergoing PAO due to hip dysplasia are registered in both the Aarhus PAO-database and DNPR. The registration completeness for the Aarhus PAO-database is therefore significantly higher than the 80% that is considered acceptable for a national registry [18]. Both registries had registered a small number of patients (2–3%) that were not found in the other registry. The high completeness indicates that these two registries are highly accurate and thus a valuable resource when investigating patients with hip dysplasia that undergo PAO. In addition, almost all patients with a discrepancy could be verified as having undergone PAO due to hip dysplasia, using the medical records. Less than 5 PAO procedures from DNPR had not undergone PAO due to hip dysplasia, and none in the Aarhus PAO-database, indicating that the Aarhus PAO-database was marginally more accurate than the DNPR. The registration completeness was 8% lower when the date of PAO was added to the analysis. Even though the median difference in date of surgery was only 3 days, among patients with more than 1 days difference, this information is important for future research investigating outcomes shortly after the operation, such as complications or days of hospitalisation.

To the best of our knowledge this is the first validation study investigating the registration completeness in a disease registry for patients with hip dysplasia undergoing PAO, despite the existence of similar registries [19, 20]. However, registries regarding other diagnoses have been validated in a similar way [21–24]. The overall completeness of registration for primary total hip arthroplasty in the Danish Hip Arthroplasty Register compared to DNPR, was 94.1% (95% CI: 93.9%;94.4%) from 1995 to 2000 among all hospitals in Denmark [21]. The registration completeness is thus similar to the registration completeness in the present study, despite a large difference in the number of sites, as The Danish Hip Arthroplasty Register includes 48 orthopaedic departments in Denmark, whereas the Aarhus PAO-database includes two, due to PAO being a highly specialised surgical procedure. In addition, the present study included both a public and a private hospital, whereas the study by Pedersen et al., excluded all patients that underwent surgery at a private hospital [21].

The Danish Knee Ligament Reconstruction Register was compared to DNPR in 2013, investigating the registration completeness of knees that had undergone reconstruction of the anterior cruciate ligaments (ACL) [22]. The overall completeness of registration was 79% (95% CI: 78;79) from 2005 to 2021, and increasing over time [22]. The authors suggest that the increase over time might be due to surgeons becoming more familiar with the registration task. This might be the reason that there were no substantial differences in the registration completeness over time in the present study, as the surgeons are likely already familiar with the registration task as the Aarhus PAO-database was created in 2010. In addition, the registration completeness in the study by Rahr-Wagner et al. was calculated as the number of knees registered in both the Danish Knee Ligament Reconstruction Register and DNPR, divided by the number of knees registered in DNPR [22]. If the same approach was used in the present study the registration completeness would be 97.0% (95% CI: 95.9%;97.9%) and thus better than the 94.7% found, however as we suspected there were discrepancies in both registries and the conservative estimate reflects this.

To investigate the Aarhus PAO-database’s value as a research resource, the number of included patients were compared to the DNPR, and further validated against the medical records, considered the gold standard for treatment information. Despite the comprehensive validation, this study has some limitations. Firstly, only patients that had undergone PAO at either Aarhus University Hospital or Mølholm Private Hospital were included. Although these are a public and a private hospital, the same surgeons operated at both sites making registration differences unlikely. Thus, the hospitals can be considered a combined or single site for registration purposes. Secondly, only patients that had undergone PAO between 2014 and 2021 were included so the validation only applies to this period. Future research should take this into consideration and preferably use data from 2014 and onwards. Thirdly, the registration completeness estimates sensitivity [25], but the specificity could not be investigated due to the study setup.

In conclusion, the registration completeness was 95% between the Aarhus PAO-database and DNPR. The Aarhus PAO-database has thus managed to include almost all patients that had undergone PAO due to hip dysplasia from 2014 to 2021. The Aarhus PAO-database therefore seems to be a valid resource for future research projects, as well as for the quality assurance and development of the treatment offered for patients with hip dysplasia.

## Data Availability

Data is provided within the manuscript.
